# Promoting Utilization of *Saccharum* spp. Genetic Resources through Genetic Diversity Analysis and Core Collection Construction

**DOI:** 10.1371/journal.pone.0110856

**Published:** 2014-10-21

**Authors:** Spurthi N. Nayak, Jian Song, Andrea Villa, Bhuvan Pathak, Tomas Ayala-Silva, Xiping Yang, James Todd, Neil C. Glynn, David N. Kuhn, Barry Glaz, Robert A. Gilbert, Jack C. Comstock, Jianping Wang

**Affiliations:** 1 Agronomy Department, University of Florida, Gainesville, Florida, United States of America; 2 College of Life Sciences, Dezhou University, Dezhou, Shandong, China; 3 Subtropical Horticulture Research Station, USDA-ARS, Miami, Florida, United States of America; 4 Everglades Research and Education Center, University of Florida, Belle Glade, Florida, United States of America; 5 Sugarcane Field Station, USDA-ARS, Canal Point, Florida, United States of America; 6 Genetics Institute, Plant Molecular and Biology Program, University of Florida, Gainesville, Florida, United States of America; 7 FAFU and UIUC-SIB Joint Center for Genomics and Biotechnology, Fujian Agriculture and Forestry University, Fuzhou, Fujian, China; National Institute of Plant Genome Research, India

## Abstract

Sugarcane (*Saccharum* spp.) and other members of *Saccharum* spp. are attractive biofuel feedstocks. One of the two World Collections of Sugarcane and Related Grasses (WCSRG) is in Miami, FL. This WCSRG has 1002 accessions, presumably with valuable alleles for biomass, other important agronomic traits, and stress resistance. However, the WCSRG has not been fully exploited by breeders due to its lack of characterization and unmanageable population. In order to optimize the use of this genetic resource, we aim to 1) genotypically evaluate all the 1002 accessions to understand its genetic diversity and population structure and 2) form a core collection, which captures most of the genetic diversity in the WCSRG. We screened 36 microsatellite markers on 1002 genotypes and recorded 209 alleles. Genetic diversity of the WCSRG ranged from 0 to 0.5 with an average of 0.304. The population structure analysis and principal coordinate analysis revealed three clusters with all *S. spontaneum* in one cluster, *S. officinarum* and *S*. hybrids in the second cluster and mostly non-*Saccharum* spp. in the third cluster. A core collection of 300 accessions was identified which captured the maximum genetic diversity of the entire WCSRG which can be further exploited for sugarcane and energy cane breeding. Sugarcane and energy cane breeders can effectively utilize this core collection for cultivar improvement. Further, the core collection can provide resources for forming an association panel to evaluate the traits of agronomic and commercial importance.

## Introduction

Sugarcane (*Saccharum* spp.) is a perennial grass, belonging to the *Poaceae* family and *Andropogoneae* tribe, which is grown widely in tropical and subtropical regions. It is the highest yielding crop worldwide [1] and accounts for approximately 75% of the world sugar production [2], [3]. In recent years, sugarcane has gained increasing attention as a biofuel crop due to its high biomass yield potential [4]. As a C4 plant, sugarcane is one of the world's most efficient crops in converting solar energy into chemical energy through photosynthesis and has a favorable energy input/output ratio [5], [6]. Besides sucrose-based ethanol production, which replaces 30% of the gasoline consumed in Brazil [7], sugarcane lignocellulosic biomass-based ethanol is an increasingly attractive biofuel to supplement fossil fuels. As a result, energy cane breeding programs have emerged and separated from sugarcane breeding programs, though both breeding programs employ interspecific hybrids from crosses between species primarily within the genus *Saccharum*. Sugarcane cultivars are selected primarily for high sucrose content and energy cultivars for high biomass and fiber with low sucrose content. Biomass level of energy cane cultivars out-performs many other grasses cultivated for biofuel production, including switchgrass, elephant grass, *Miscanthus*, and sorghum in the southern US [8], [9]. Thus, energy cane is suited for lignocellulosic ethanol production while sugarcane can be used for sucrose ethanol production as in Brazil.

The origin of modern sugarcane cultivars is from inter-specific hybridizations of domesticated species *S. officinarum* (2n = 80, x = 10) which is characterized by high sugar and low fiber content [10] and the wild species *S. spontaneum* (2n = 40–128, x = 8), which is resistant to biotic and abiotic stresses [11]–[13]. Modern sugarcane genotypes are highly polyploid and aneuploid with multiple alleles at each locus. The genome composition of sugarcane cultivars has been estimated as 85% from *S. officinarum* and 15% from *S. spontaneum* [14]. The genome complexity in *Saccahrum* spp. has made sugarcane and energy cane breeding cumbersome. The genotypes utilized over decades in earlier breeding programs are a limited number of *S. spontaneum* and *S. officinarum* clones, which has resulted in a narrow genetic base of sugarcane cultivars [15]. Hence, it is important to characterize the genetic variation among the domestic cultivars and the available genetic resources in order to exploit them and accelerate sugarcane and energy cane improvement. A germplasm collection with high genetic diversity would enable breeders to broaden the genetic base of parental lines and thereby facilitate genetic gains of sugarcane and energy cane cultivars [16], [17].

The classification of the *Saccharum* spp. based on morphology, chromosome numbers and geographic distribution has been a matter of debate for a long time. The *Saccharum* genus was believed to consist of six major species, including two wild species *S. spontaneum* and *S. robustum* and four cultivated species, *S. officinarum*, *S. barberi*, *S. sinense* and *S. edule* [18], [19]. However, there were controversial reports by Irvine 1999 mentioning the existence of only two *Saccharum* species: *viz. S. officinarum* and *S. Spontaneum* [19]. The *Saccharum* genus together with related genera, such as *Erianthus*, *Miscanthus*, *Narenga*, and *Sclerostachya* were referred to as the “*Saccharum* Complex” [20]. However, there are limited attempts to characterize the *Saccharum* complex using molecular markers [21], [22]. There is a need to trace the domestication and evolution of *Saccharum* spp by extensive molecular dissection. Two duplicated “*Saccharum* Complex” germplasm collections known collectively as the “World Collection of Sugarcane and Related Grasses” (WCSRG) were utilized. One WCSRG is maintained in Coimbatore, India and the other in Miami, FL, USA. The National Germplasm Repository located at the USDA-ARS Subtropical Horticulture Research Station in Miami, FL maintains the WCSRG in the USA [23], [24]. This WCSRG may contain significant genetic diversity and many valuable alleles for numerous morphological traits, biomass yield components, adaptations to biotic and abiotic stresses, and many other quality traits [25]. Earlier studies on genetic diversity analysis in selected clones in this collection have provided limited information [26], [27]. In addition, limited numbers of clones in the WCSRG have been used for sugarcane and energy cane improvement. This large genetically diverse collection with vast potential remains unutilized.

With its large number and genetically complex accessions, it is a formidable task to fully characterize and use the WCSRG in breeding programs. A core collection that is a condensed assembly of the entire collection with maximized genetic diversity and minimized redundancy is essential for its utilization [28]. Such a core collection for *Saccharum* spp. would provide a subset of representative accessions and can facilitate extensive examination at phenotypic, physiological and genetic levels. Thus, it could substantially utilize the contributions of the WCSRG in sugarcane and energy cane breeding programs.

Genetic markers are widely applied for diversity analysis, genetic trait mapping, association studies and marker assisted selection [29]. Simple sequence repeats (SSR) or microsatellites [30] are tandem repeats of 1 to 6 base pairs of DNA, which are found in all eukaryotic genomes [31], [32]. During the last decade, SSR markers have been powerful tools for diversity assessment of populations in many crops including *Zea mays* [33], *Sorghum bicolor* [34], *Solanum lycopersicum* [35], *Oryza sativa* [36], *Vitis* [37], *Triticum aestivum* [38], *Hordeum vulgare* [39] and *Eucalyptus* [40]. In sugarcane, SSRs have been used for germplasm evaluation [41]–[45], QTL analysis and genetic map development [46]. Thousands of SSR markers located randomly in the sugarcane genome available in public domain [27], [47], [48] provide an essential tool for genotyping. Our objectives were to genotypically evaluate all the 1002 accessions in WCSRG germplasm using SSR markers and to understand the genetic diversity and population structure of this collection and create a core collection of 300 accessions that captures the vast majority of genetic diversity present in the larger collection for further utilization in breeding programs.

## Materials and Methods

### Plant materials

**Figure 1:**
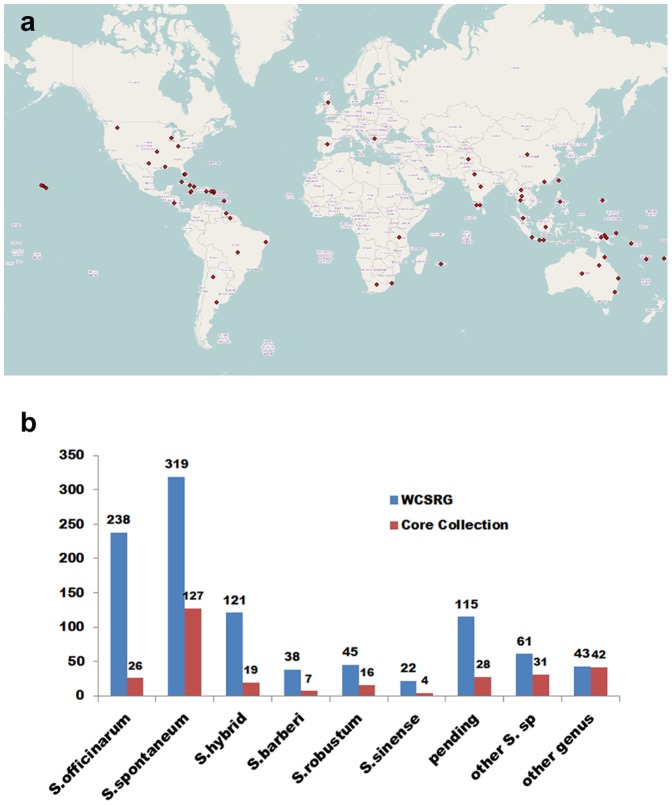
Distribution of the World Collection of Sugarcane and Related Grasses (WCSRG). (a) Geographic distribution of the accessions in the WCSRG. The 1002 accessions in the WCSRG were obtained from 45 countries. Each red dot represents a sugarcane collecting location. Global Mapper V14 software with OpenStreetMap was used to locate the accessions based on the latitude and longitude of origins. (b) Numerical distribution of the different species in the WCSRG and the core collection identified.

The WCSRG is part of the USA National Plant Germplasm System (NPGS) (http://www.ars-grin.gov/npgs/index.html). The NPGS caters the need of researchers by acquiring, preserving, evaluating, documenting and distributing crop germplasm. There were 1002 non-redundant accessions in the WCSRG maintained at the USDA-ARS Subtropical Horticulture Research Station, Miami, FL, and made available for free distribution. These accessions were mostly survivors from Hurricane Andrew in 1992 with some curated new accessions. The *S. spontaneum* accessions are maintained in 7-gallon pots on a concrete pad and not allowed to flower as they are considered invasive. The rest of the accessions are planted in the field and rotated to new field plots every 4 years. The mature plants are cut to the ground every year in the early spring until replanting. The accessions represent collections from 45 different countries (Fig. 1a). *Saccharum officinarum*, *Saccharum* hybrids and *S. spontaneum* comprised the major portion of the collection and minor portion includes the other species such as *Coix gigantea*, *Imperata* spp., *Miscanthus floridulus*, *Miscanthus* hybrids, *Miscanthus sinensis*, *Miscanthus* spp., *Narenga porphyrocoma*, *Saccharum arundinaceum*, *Saccharum barberi*, *Saccharum bengalense*, *Saccharum brevibarbe*, *Saccharum edule*, *Saccharum* hybrids, *Saccharum kanashiroi*, *Saccharum officinarum*, *Saccharum procerum*, *Saccharum ravennae*, *Saccharum robustum*, *Saccharum rufipilum*, *Saccharum sinense*, *Saccharum spontaneum*, *Saccharum* spp., *Sorghum plumosum*, *Sorghum arundinaceum* and some unknown or pending accessions (Fig. 1b, Table S1). The species name of each accession in the WCSRG was defined based on the curator’s naming system. Young leaf tissues of these 1002 accessions were collected in 2011 and lyophilized for DNA isolation.

### DNA extraction and PCR conditions

The genomic DNA was extracted from 500 mg lyophilized leaves using the CTAB method according to Wang et al [45] with minor modifications. The quality and quantity of the genomic DNA was checked using 1% agarose gel electrophoresis by comparison with a known concentration of lambda DNA as a standard (New England). The DNA with good quality was then diluted to 1.25 ng/µl for the PCR.

PCR reactions were carried out in a 10 µl volume containing 2.5 ng genomic DNA, 1 × PCR buffer, 25 mM MgCl_2_, 2 mM dNTP, 2 µM of each primer, and 1 U Taq polymerase. The reaction was performed in an ABI thermal cycler with the following cycling condition: 94 °C for 3 min; followed by 35 cycles of 94 °C for 30 s, then the appropriate annealing temperature for 30 s, 72 °C for 30 s, followed by one cycle at 72 °C for 7 min. The annealing temperature for each primer was optimized separately and ranged from 46 °C to 64 °C (Table S2).

### SSR genotyping

In total, 191 SSR primer pairs selected from different publications (Table S2) were screened on a panel of eight diverse genotypes belonging to *S. robustum, S. arundinaceum*, *S. officinarum*, *S. spontaneum* and *S.* hybrid to select the SSR markers with high polymorphic information content (PIC). The selected SSR markers were then used for genotyping each accession in the WCSRG.

Two genotyping platforms, polyacrylamide gel electrophoresis (PAGE) with silver staining and capillary electrophoresis with an ABI 3730 sequencer were used to separate/visualize the PCR products. For the PAGE system, a C.B.S. electrophoresis unit (C.B.S Scientific Co. Del Mar, CA) was used for the PCR product separation. The amplified products were loaded in non-denaturing 6% polyacrylamide gel electrophoresis [160.2 mL 0.5931X TBE buffer, 28.5 mL 40% acrylamide/bis-acrylamide solution [19∶1 (w/v)], 1.33 mL 10% APS (ammonium persulfate), and 66.5 µl TEMED]. The electrophoresis was conducted in 0.5 X TBE running buffer at 350 V for approximately 1 hour 45 minutes and SSR amplicons were visualized by silver staining (0.2% AgNO_3_) according to the modified protocol of Creste et al. [49]. The size of each allele was determined by comparing it to the 100 bp DNA ladder (New England Biolab INC.). The robust bands were scored as present (1) or absent (0) and a score file binary matrix (0/1) was used for further analysis.

For the ABI 3730 sequencer system, forward primers were labeled with fluorescent dyes, 6-FAM, VIC, NED or PET, allowing subsequent multiplexing. PCR reactions of the four primer pairs were performed independently, and the amplified PCR products were checked on a 1% agarose gel. The optimized amounts of four different fluorescence dye-labeled PCR products of the same genotype were multiplexed. Combined PCR products were denatured at 95 °C for 5 min and mixed with GeneScan™ 600 LIZ™ size standard (Applied Biosystems, USA) and Hi-Di formamide for separation on ABI 3730 Genetic Analyzer (Applied Biosystems, USA). The GeneScan files generated were analyzed using GeneMarker V2.4.0 (Softgenetics, LLC, State College, PA, USA). The peak sizes were automatically calibrated against the 600 LIZ™ size standards with default module settings. The alleles were mainly called by the GeneMarker software in couple with manual rechecking. The presence of a peak was scored as “1” and its absence was designated as “0”. The genotypic data are made publically available through the Germplasm Resources Information Network (GRIN) database (http://www.ars-grin.gov/), which has an open free access to scientists in the world-wide community, and will be available upon request.

### Genetic diversity analysis

The binary data matrix of alleles for each SSR locus was constructed from evaluation of all the accessions in the WCSRG. PowerMarker V3.25 software was used to calculate allele frequency, number of alleles per locus, percentage of polymorphic bands, PIC, and gene diversity (expected heterozygosity, *He*) [50]. Shannon’s Information Index of Diversity (I) and Nei’s distance were estimated for pre-defined species by GenAlEx Ver 6.5 [51]. The probability of identity [52] and the power of exclusion [53] were calculated using allele frequencies from the 1002 accessions. Cluster analysis was carried out using DARwin V5.0.137 software [54]. A dissimilarity matrix was calculated by considering Dice coefficient with pairwise variable deletion. The dissimilarity matrix was used to generate a phylogenetic tree by using the Neighbour-joining (NJ) method with 500 bootstrap replicates. For selection of core collection, the Maximization (M) algorithm implemented in DARwin software was applied with the highest genetic diversity. The Principal Coordinate Analysis (PCoA) was generated based on the Genetic Distance matrix by GenAlEx Ver 6.5 [51].

### Population structure and differentiation analysis

The population structure and number of subpopulations present in the WCSRG was assessed by model-based clustering algorithms using STRUCTURE V2.2 [55]. The number of subpopulations (*K*) was set from 1 to 15, and at least ten runs per K were conducted separately with 100,000 generations of ‘burn-in’ and 100,000 Markov chain Monte Carlo (MCMC). The best K value was determined based on ad hoc quantity (ΔK) analysis [56]. Analysis of Molecular Variance (AMOVA) was conducted to detect the genetic variance within and among WCSRG subpopulation using GenAlEx Ver 6.5 [51].

## Results

### SSR genotyping

A pilot experiment was carried out for screening 191 sugarcane SSR markers (Table S2) with eight *Saccharum* accessions belonging to different species. These markers yielded 276 alleles with 2–13 alleles per primer pair and their PIC value ranged from 0.195 to 0.375. To screen WCSRG, 36 SSR markers with high PIC values were selected to genotype each accession in the WCSRG. Out of 36 SSR markers, 14 primer pairs could be located on eight different sorghum chromosomes and the other 22 could not be mapped on sorghum genome (Table S2). In total, 209 alleles, which constituted 100 from PAGE and 109 from capillary electrophoresis, were recorded among the 1002 accessions with an average of 5.8 alleles per locus. The number of alleles recorded per locus ranged from 1 at UGSuM349 to 17 at UGSM667. The highest number of alleles, 13 and 17 were found at locus SCA10 and UGSM667 respectively (Table 1). In total, 5–12 alleles were observed at 18 SSR and 3 or fewer alleles at 10 SSR loci. SSRs having di-nucleotide repeats were more polymorphic than other repeat motifs (Table S2). Of the 36 primer pairs, 21 were screened on the PAGE platform and 15 were screened by capillary electrophoresis on the ABI 3730 sequencer platform. In order to compare the results of both platforms, some labeled primers screened by the ABI 3730 were checked on the PAGE platform and the results were comparable in terms of molecular weight of the amplicons.

**Table 1:** Parameter list of 36 simple sequence repeat (SSR) primer pairs used for genotyping of 1002 accessions in the World Collection of Sugarcane and Related Grasses in Miami, FL.

Primer name	Product size (bp)	Alleles (No.)	Major allele frenquency	Range of PIC^a^ values	Mean of PIC values	*I* ^b^	Q^c^
SCAA8L15-12A	180–400	11	0.996	0.008–0.372	0.168	0.028	0.750
SC109D21-11-2	100–1000	8	0.918	0.102–0.375	0.317	0.038	0.706
SC159C20-21a	150–700	4	0.984	0.062–0.368	0.232	0.125	0.474
SMC24DUQ	140–150	3	0.975	0.048–0.327	0.166	0.188	0.367
SMC334BS	160–190	6	0.913	0.157–0.375	0.292	0.069	0.604
SMC569CS	180–300	4	0.959	0.076–0.273	0.192	0.291	0.281
SMC22DUQ	150–180	4	0.911	0.153–0.340	0.229	0.137	0.452
mSSCIR43	180–280	11	0.922	0.136–0.374	0.283	0.030	0.742
SMC31CUQ	120–500	2	0.567	0.371–0.372	0.372	0.375	0.187
SCA09	90–400	4	0.977	0.163–0.370	0.268	0.147	0.438
SCA10	200–250	15	0.997	0.006–0.374	0.240	0.016	0.811
SCB10	100–500	4	0.989	0.022–0.190	0.129	0.110	0.503
SCM21	160–450	3	0.940	0.136–0.334	0.256	0.189	0.366
UGSM60	200–900	5	0.909	0.123–0.343	0.220	0.073	0.592
UGSM585	160–1000	6	0.981	0.095–0.359	0.252	0.054	0.651
UGSM667	160–1000	17	0.984	0.029–0.375	0.272	0.012	0.840
SEGM2dot	180–100	2	0.747	0.307–0.369	0.337	0.395	0.178
SEGM285	290–800	8	0.873	0.197–0.374	0.315	0.042	0.692
UGSM459	380–600	10	0.989	0.022–0.374	0.263	0.036	0.716
UGSM594	200–700	3	0.928	0.143–0.360	0.246	0.339	0.229
UGSM629	300–1000	4	0.902	0.248–0.336	0.272	0.189	0.377
UGSM694	20–1000	3	0.883	0.217–0.292	0.244	0.187	0.369
UGSM399	120–600	3	0.871	0.199–0.373	0.314	0.205	0.348
UGSuM26	180–800	3	0.952	0.081–0.185	0.144	0.186	0.370
UGSuM349	400–1000	1	0.610	0.363	0.363	–	–
UGSuM43	250–1000	5	0.922	0.145–0.250	0.205	0.072	0.595
UGSuM15	220–420	3	0.921	0.134–0.353	0.278	0.240	0.314
UGSuM186	130–500	4	0.791	0.293–0.374	0.352	0.125	0.474
UGSuM197	120–500	9	0.998	0.002–0.374	0.171	0.064	0.620
UGSuM337	200–550	11	0.997	0.006–0.367	0.176	0.017	0.806
UGSuM97	120–380	6	0.690	0.360–0.375	0.357	0.055	0.647
UGSuM96	150–480	5	0.993	0.014–0.354	0.279	0.092	0.547
UGSuM56	120–800	4	0.968	0.059–0.348	0.241	0.189	0.381
UGSuM34	180–700	5	0.952	0.194–0.375	0.278	0.146	0.441
UGSuM21	500–900	7	0.937	0.014–0.354	0.279	0.065	0.618
UGSuM45	210–500	6	0.933	0.059–0.348	0.241	0.095	0.541

a PIC, polymorphism information content; ^b^ *I*, Probability of identity; ^c^ *Q*, Power of exclusion.

### Allele frequency and genetic diversity in the WCSRG

**Figure 2:**
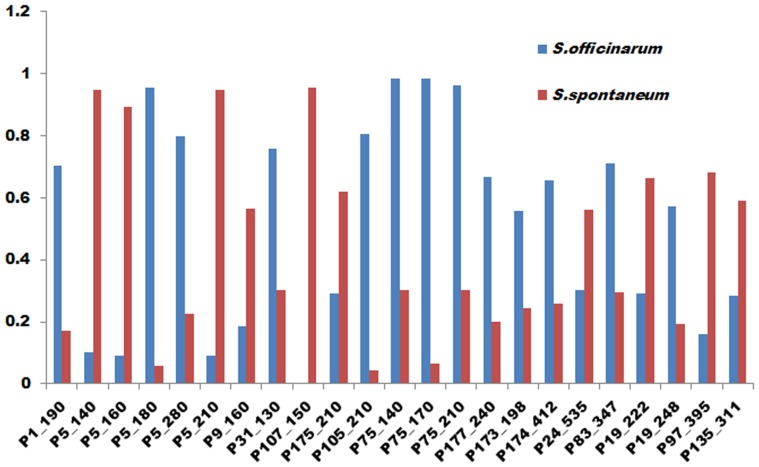
Comparison of frequencies of 23 alleles in *S. officinarum* and *S. spontaneum*. These alleles were selected based on the presence of the prevalent allele in any of the major species. For instance, presence of alleles in at least 30% of the cases in *S. officinarum* and at least 55% of the cases in *S. spontaneum*.

Major allele frequency ranged from 0.567 to 0.998 with a mean of 0.911 (Table 1). The mean PIC value of each SSR marker ranged from 0.1294 to 0.3717 with an average of 0.2568. The probability of identity (*I*) was low in most cases. It ranged from 0.012 (UGSM667) to 0.395 (SEGM2dot) with an average of 0.132. For the majority of primer pairs, the power of exclusion (Q) was moderate ranging from 0.178 (SEGM2dot) to 0.840 (UGSM667) with an average of 0.515 (Table 1). Out of the 209 alleles, 23 alleles showed significantly different frequency between the two major species, *S. spontaneum* and *S. officinarum,* with 10 alleles more frequently observed in *S. spontaneum* than in the other species. Allele UGSM629_150 was observed solely in *S. spontaneum* (Fig. 2). The highest percentage of polymorphic bands (99.52%) was found in *S. spontaneum* followed by *S. officinarum* (95.22%) and *S. robustum* (85.65%) (Table 2). The average Shannon’s Information Index scores for *S. spontaneum*, *S. officinarum*, *S.*hybrid, *S. barberi*, *S. robustum*, and *S. sinense* were 0.492, 0.456, 0.452, 0.423, 0.427 and 0.383 respectively (Table 2) indicating *S. spontaneum* is genetically more diverse than the other species. The gene diversity of each allele ranged from 0.002 to 0.500 with an average of 0.310. Among the six major pre-defined species, the highest gene diversity was found in *S. spontaneum* (0.306) followed by *S. robustum* (0.263), with an average of 0.276 (Table 2). Based on the Nei’s genetic distance, the largest genetic distance (0.079) was between *S. spontaneum* and *S. officinarum*, and the smallest (0.013) between *S. officinarum* and *S.* hybrid and other *S.* spp. with unknown accessions (Table 3).

**Table 2:** Gene diversity, Shannon’s information index, and polymorphism status of six species of *Saccharum* and other categories.

Species	Gene diversity	I^a^	Polymorphic bands (%)
*S. officinarum*	0.2564	0.456	95.22
*S. spontaneum*	0.3032	0.492	99.52
*S.* hybrid	0.2531	0.452	93.30
*S. barberi*	0.2398	0.423	85.17
*S. robustum*	0.2670	0.427	85.65
*S.sinense*	0.2381	0.383	75.60
pending	0.2985	0.486	96.65
other *S. sp*	0.2756	0.462	91.39
other genus	0.3030	0.4470	91.39

a Shannon’s Information Index.

**Table 3:** Genetic distance between six species of *Saccharum* and three other categories of species.

	*S. officinarum*	*S. spontaneum*	*S.* hybrid	*S. barberi*	*S. robustum*	*S. sinense*	unknown	other *S. spp*	other genus
*S. officinarum*	0								
*S. spontaneum*	0.079	0							
*S.* hybrid	0.013	0.071	0						
*S. barberi*	0.03	0.064	0.026	0					
*S. robustum*	0.023	0.058	0.018	0.034	0				
*S. sinense*	0.038	0.074	0.031	0.023	0.027	0			
unknown	0.021	0.037	0.019	0.03	0.016	0.034	0		
other *S. spp*	0.047	0.042	0.039	0.046	0.026	0.048	0.013	0	
other genus	0.071	0.045	0.065	0.07	0.043	0.074	0.023	0.009	0

### Phylogeny and population structure of the WCSRG

**Figure 3:**
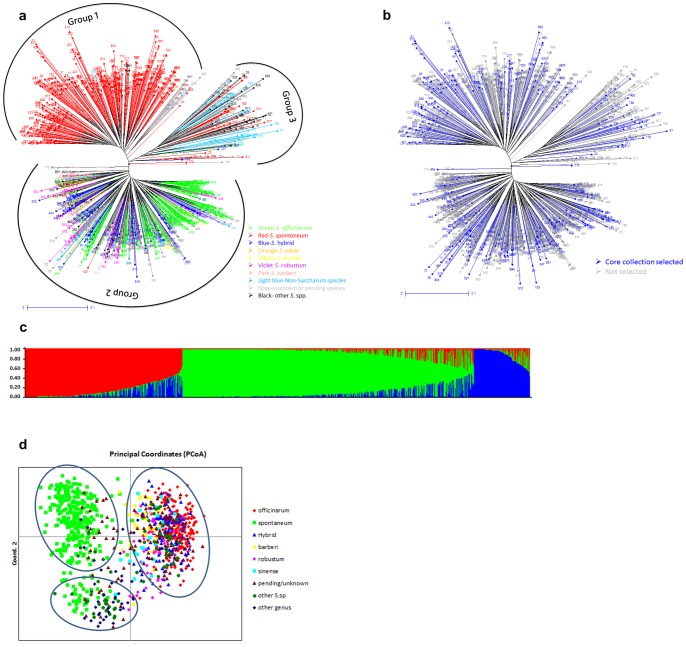
The clusters of the World Collection of Sugarcane and Related Grasses (WCSRG) and core collection in Miami, FL USA. (a) Phylogenetic tree of the WCSRG using neighbor-joining analysis. (b) Representativeness of the 300 accessions (colored blue) of the core collection selected from the WCSRG. Accessions not selected for the core collection are shaded grey. (c) The population structure of the WCSRG based on model-based estimation of 209 alleles. The WCSRG is grouped into three subgroups. Each individual is represented by a vertical line. Each color represents one subpopulation, and the length of the colored segment shows the proportion of membership for that accession. (d) Two-dimensional plot of the distribution of the WCSRG through principal coordinate analysis based on genetic distance generated from 209 alleles. The different colors represent nine pre-defined species.

Genotypic data of 209 alleles on the 1002 accessions were used to analyze the genetic distance between each accession. The phylogenetic tree of the WCSRG revealed three major clusters (Fig. 3a). All the accessions in *S. spontaneum* clustered in group 1, *S.* hybrids clustered with *S. officinarum*, *S. robustum, S. barberi, S. edule* and *S. sinense* in group 2 while the majority of accessions of unknown speciation and the species in other genera such as *Erianthus*, *Miscanthus*, and *Sorghum* (Fig. 3a) clustered in group 3. The PCoA of the WCSRG also revealed three groups and the first three axes together explain 15.20% of cumulative variation. In the PCoA plot, the first and second principal coordinates account for 7.88% and 12.54% of the total variation respectively (Fig. 3d).

The population structure of the WCSRG was analyzed by STRUCTURE V2.2. The ad hoc quantity (ΔK) analysis [56] shows a clear peak at K = 3, revealing the presence of three subpopulations in the WCSRG (Fig. 3c). Of the 1002 accessions, the 731 were clearly assigned to three specific subpopulations with membership probability greater than 0.8 and the remaining 271 accessions were an admixture subpopulation with membership probability <0.8. Subpopulation 1 comprised accessions from *S. spontaneum* and subpopulation 2 consists mostly of accessions from *S. officinarum*. The subpopulation 1 had 211 accessions including two accessions (41–158,45–19) of uncertain species name, which are likely *Spontaneum* spp. Subpopulation 2 consisted of 218 *S*. *officinarum* accessions, 101 *S.* hybrid accessions, 66 accessions from other *Saccharum* species, 4 *Miscanthus* hybrid accessions, and 55 unknown/pending accessions. Seventy-six accessions were identified in subpopulation 3 including two major non-*Saccharum* species existing in the WCSRG such as *Miscanthus* and *Erianthus*, which show high genetic divergence compared with subpopulations 1 and 2.

The distance-based AMOVA analysis revealed genetic variance among and within the populations were highly significant (P<0.001) and the variation within subgroups (89%) was significantly higher than that among subgroups (11%) (Table 4). Significant variance not only exists among three major subpopulations inferred by the structure analysis but also among six major *Saccharum* species, which were pre-defined by the germplasm curators. However, based on the AMOVA analysis, the фst (0.160) among the three major subpopulations inferred by the structure analysis was higher than the фst (0.108) among the six major species.

**Table 4:** Analysis of molecular variance (AMOVA) among 9 pre-defined populations and three structure detected populations within World Collection of Sugarcane and Related Grasses (WCSRG).

Population	Source of variation	Degrees of freedom (df)	Sum of squares	Mean sum of squares	Estimated variance	Percentage of variation (%)
9 pre-defined populations	Among Pops	8	3432.803	429.1	3.935	11
	Within Pops	993	32121.42	32.348	32.348	89
	Total	1001	35554.22		36.283	100
	Fixation Index	Фst = 0.108				
3 Structure detected populations	Among Pops	2	3476.653	1738.326	6.11	16
	Within Pops	999	32077.78	32.11	32.11	84
	Total	1001	35554.44		38.22	100
	Fixation Index	Фst = 0.160				

### Constructing a core collection

To construct a core collection representing most of the genetic diversity in the WCSRG, the maximum length sub-tree for disequilibrium was calculated using DARwin. From this, a core collection of 300 accessions representing most of the genetic diversity was identified (Fig. 3b). Genetic diversity analyses showed that the average major allele frequency of the core collection was 0.75, which is comparable to the value of 0.77 calculated for the WCSRG. Similarly, gene diversity was 0.337 with the range from 0 to 0.5 in the core collection, which was comparable to 0.304 in the WCSRG. The PIC value of the alleles was 0.269 in the core collection and 0.245 in the WCSRG. Genotype frequency of the core collection and the WCSRG were both 0.5 (Table 5). These results indicated that the core collection adequately represents the genetic diversity of the WCSRG.

**Table 5:** Diversity parameters of the World Collection of Sugarcane and Related Grasses (WCSRG) in Miami, FL and the core collection of sugarcane and related species.

Diversity parameters	WCSRG (1002 accessions)			Core collection (300 accessions)		
	Max	Min	Mean	Max	Min	Mean
Major allele frequency	0.9990	0.5060	0.7747	0.9990	0.5050	0.7488
Gene Diversity	0.4999	0.0020	0.3041	0.4999	0.0067	0.3371
PIC of alleles	0.3750	0.0020	0.2450	0.3750	0.0067	0.2690
Genotype frequency	0.9990	0.0010	0.5000	0.9997	0.0034	0.5000
Co-variance	0.0003	0.0000	0.0002	0.0009	0.0000	0.0006

## Discussion

Genotypic evaluation of the sugarcane germplasm as a potential breeding material provides essential information so that cane breeders can utilize more genetically diverse parents in their breeding programs. In this study, we evaluated all 1002 accessions available in the WCSRG using SSR markers to estimate the genetic diversity and select accessions for the core collection. The WCSRG is currently not widely used but is potentially a great resource for sugarcane and energy cane breeders to improve commercial cultivars. We report here the results of the first extensive genetic diversity study on all accessions available in the WCSRG maintained in USA. With this information, sugarcane and energy cane breeders will now have information on the WCSRG that will allow them to make long-term improvements of commercial cultivars with important agronomic traits.

Because sugarcane is extremely heterozygous and highly polyploid, polymorphisms are high among the accessions. Analysis of SSR markers on the WCSRG indicated 1 to 17 robust polymorphic alleles with an average of 5.8 alleles per locus, comparable to other studies, where the allele number per locus was 7.35 [57] and 8.78 per locus [58]. Perhaps the slightly lower number of alleles per locus reported in this study was due to the higher stringency applied in allele scoring. Of the 36 SSR loci, 14 were aligned to different chromosomes of sorghum whereas the other 22 had no similarity to the sorghum genome (Table S2). These 22 SSR loci are most likely located in non-coding regions of the sugarcane genome where the sequences are highly diverged from those of the sorghum genome. In light of the synteny between the sorghum and sugarcane genome [48], [59], these 36 SSR loci should cover the sugarcane genome randomly, therefore, the sugarcane genome was sampled randomly by the 36 SSR loci for the phylogenetic study of the WCSRG. In addition to SSR markers, Chandra et al. developed conserved-intron scanning primers (CISP) could be a choice to evaluate the polymorphic potential in sugarcane and related species and reveal the relationships among sugarcane germplasm [60].

The probability of identity (*I*) is an individual identification estimator which explains the probability of two different accessions having the same genotypes at one specific locus in a population by chance rather than through inheritance. It was calculated based on the allele frequencies for each marker from the WCSRG. The *I* values ranged between 0.012 (UGSM667) and 0.395 (SEGM2dot) (Fig. 1b). For most of the SSRs used in this study, the *I* values were low and the combined probability for all markers was 9×10^−37^ indicating that the 36 markers are capable of distinguishing all accessions in the WCSRG. The exclusion probability (*Q*) indicates the probability of excluding an accession from the possibility of parentage if the accession was not involved in any parentage. The *Q* values were moderate for most SSR primers, ranging from 0.178 (SEGM2dot) to 0.840 (UGSM667) (Table 1). The combined power of exclusion exceeded 99.99%, which indicates that these SSR markers were able to discriminate among all of the accessions with nearly a 100% probability of excluding any false parentage.

The presence of 20 significantly different alleles between *S. spontaneum* and *S. officinarum* suggests genomic differences, which could act as gene flow barriers between them. The species-specific alleles were also found [61] using maize SSRs, where they identified five alleles specific to *Erianthus*, *S. spontaneum* and *S. officinarum*. These alleles can be used to detect genome components of *S. spontaneum* in the hybrids.

Classification of the *Saccharum* species has been a topic of debate for many years. The *Saccharum* genus was traditionally divided into six species: *S. spontaneum*, *S. officinarum*, *S.robustum*, *S. edule*, *S. barberi* and *S. sinense*, which were defined by some highly variable characters with many uncertainties [18], [19]. However, Irvine [19] considered them as two species: *S. spontaneum* and *S. officinarum* with the other four species and hybrids being considered as *S. officinarum* based on the morphological, cytological and genotypic analysis. In this study, phylogenetic analysis based on genetic diversity indicated that accessions of *S. spontaneum* clustered into a major group/subpopulation. *S. officinarum* along with other *Saccharum* species such as *S. sinense, S. barberi*, *S. robustum, S.* hybrids and other genus *Narenga* were clustered into another distinctive group/subpopulation (Fig. 2a, 2c, Table S3), indicating the close relationship among these species, which should be considered as one species specifically given the non-barrier intercrossing nature among them. The third group comprised of the genotypes from other genus like *Coix*, *Miscanthus* and some *Saccharum* species as named by the curators such as *S. bengalense*, *S. arundinaceum*, *S. ravannae*, *S. procerum*, *S. brevibarbe* and *S. rufipilum.* Based on phylogenetic analysis, *S. bengalense*, *S. arundinaceum*, *S. ravannae* and *S. procerum* should be named as *Erianthus* species such as *E. bengalense*, *E. arundinaceum*, *E. ravannae* and *E. procerum* respectively (Table S1). This concurred with predecessor research results [62]. *Saccharum brevibarbe* and *S. rufipilum* should be considered as non-*Saccharum* species since they were distinctively clustered in the non-*Saccharum* group. Interestingly, several designated *Erianthus unknown* clones were found in group 2 clustered with the *S. officinarum*, which might be *Saccharum* spp. and need to be further validated.

The classification of the WCSRG through phylogenetic analysis revealed three groups (Fig. 3a), which corresponds with three subpopulations identified by population structure analysis (Fig. 3c). The subpopulation 1 contained the majority of *S. spontaneum* with the membership probabilities of >0.80, almost all the *S. officinarum* and hybrids assigned to subpopulation 2, and within subpopulation 3, non-*Saccharum* species, including *Erianthus* and *Miscanthus* along with some unknown species, share membership with a few *S. spontaneum* accessions (Table S3). These results indicate that the *Saccharum* species should be classified into two major species: *S. spontaneum* and *S. officinarum* and this supports the findings of Irvine [19]. The higher фst value of 0.160 among the three major subpopulations inferred by the STRUCTURE analysis compared with the фst value of 0.108 among the six pre-defined major species along with three other categories also supports the conclusion that there are only two major *Saccharum* species (Table 4). Hodkinson et al. [63] used three DNA sequences to study the inter-relation between *Miscanthus*, *Saccharum* and other related genera and found that there was polyphyletic relationship between *Saccharum* spp. and *Miscanthus* spp. Most interestingly, the species known to be *Saccharum* complex (*S. ripidium*) did not group closely with any of the *Saccharum species* and there was no evidence of division of *Saccharum* into *Erianthus and Narenga* [63]. Cai et al. [21] investigated the genetic diversity within the “*Saccharum* complex” and indicated *Saccharum* spp. are grouped together and are apart from non-*Saccharum* spp. Similar results were observed in WCSRG in this study (Fig. 3a and 3c). The species name of each accession in the WCSRG was defined based on the curator’s records or geography and the species identities of some accessions were unknown. The genetic diversity analysis and genetic structure of the WCSRG will not only assist us in efficient utilization of germplasm but also in identifying the species of some of these unknown accessions in the collection. The study also provides the genetic information about the mis-designated species, which can be used to correct the taxonomic classification after proper validation.

*Saccharum spontaneum* having high genetic variability is used extensively in sugarcane and energy cane breeding programs to provide tolerance and resistance to a wide range of biotic and abiotic stresses. Among *Saccharum* species, *S. spontaneum* is thought to have the widest ecogeographical distribution and the highest variation for chromosome number 2n = 40–128 [11]. *Saccharum officinarum* is the closest relative with modern sugarcane cultivars which contain approximately 80–85% of the genetic background of *S. officinarum* [14], [64]. Hence, hybrids in the germplasm collection have a closer relationship with *S. officinarum* than with *S. spontaneum.* The phenotypic characters of the same populations showed the similar clustering with *S. spontaneum* grouping separately from most of other *Saccharum* spp [65]. This corroborates with our genotypic data on the division of the populations indicating that this genotypic diversity does correlate with physical traits and phenotypic diversity and could be useful to breeders [65].

A core collection selected from the entire germplasm collection is of the utmost importance for breeders and geneticists working to improve sugarcane and energy cane. A number of studies have been carried out to construct a representative core collection in many crop plants because of the availability of a large germplasm collection, such as in *Oryza sativa* [36], *Sorghum bicolor* [66], and *Zea mays* [67], [68]. Several efforts have been invested in constructing core collections from *S. officinarum* [69] and *S. spontaneum* [70] separately based on the phenotypic evaluations. For instance, 716 accessions of *S. officinarum* maintained in India [69] were evaluated for 37 phenotypic and morphological descriptors like leaf length, leaf shape, internode angle, ligule shape, Brix content, etc. A core collection of 185 accessions was derived in accordance with the diversity in the 716 accessions based on principal component scores and the Shannon-Weaver Diversity Index [69]. Tai and Miller evaluated 342 *S. spontaneum* accessions maintained at the USDA-ARS, SHRS in Miami, FL for 11 phenotypic traits stalk diameter, time of flowering, leaf length, fiber content, Brix and six other traits with 11 different sampling methods. As a result, a core collection comprising of 75 clones was selected based on stratified random sampling and principle component analysis [70]. The WCSRG was phenotypically evaluated to form the core collection and there was only a portion of accessions shared between the core collections based on phenotypic data and based on genotypic data [65]. Further comprehensive analysis of both phenotypic and genotypic data by weighing the different parameters is expected to refine the core collection for *Saccharum* spp.

The core collection identified in this study consisted of 300 genotypes (29.7% of the WCSRG) including major *Saccharum* species, unknown/pending and most non-*Saccharum* spp. It will be a much more reasonable task to thoroughly characterize the reduced number of accessions and then effectively utilize them in breeding programs to broaden the genetic base of commercial cultivars. In addition, the core collection can serve as a diversity panel for marker-trait association analysis to identify alleles for important agronomic traits. The core collection has been successfully used as a panel to study association mapping for yield and grain quality traits in rice [71] and maturity and plant height in the sorghum mini-core collection [72]. In another study, eight subpopulations were identified from a panel of 154 clone using AFLP and SSR marker systems [73]. Association mapping was carried out on a set of 480 clones of sugarcane using the DArT platform and a large number of markers were found to be associated with cane yield and sucrose content [74]. Inevitably, variable structure and size could be existing in different types of core collections. The core collection generated in our study will be further refined according to phenotypic evaluation and structure effect correction to form a balanced diverse panel for the future association mapping studies.

In summary, 1002 accessions in the WCSRG maintained by the USDA in Miami, FL, USA were evaluated with 209 polymorphic alleles from 36 SSR markers. Diversity analysis showed that the WCSRG has a gene diversity of 0.304. The result from phylogenetic and structure analysis of the 1002 accessions revealed three major groups with significant differentiation among them. Based on the genotypic data, a core collection of 300 accessions was selected representing the majority of diversity in the WCSRG. The core collection developed and the data from this study provide valuable breeding resources to the sugarcane and biomass feedstock communities. These clones can be utilized for creating mapping populations that will be useful to develop QTLs and to understand the genetic basis. The information can be exploited in mapping of genes and QTLs for marker assisted introgression of traits into elite breeding lines. This characterized diverse genetic resource can be further exploited by breeders to improve both sugarcane and energy cane in *Saccharum* spp.

## Supporting Information

Table S1 **Numerical distribution of the different species in the World Collection of Sugarcane and Related Grasses (WCSRG).** Note: Asterisk (*) indicate the genus name of each accession in the WCSRG was listed based on the curator’s records. However, they are supposed to be named as non- *Saccharum* species according to our experiment results.
(XLSX)

Table S2 **Summary information of 191 simple sequence repeat (SSR) markers used to genotype 1002 accessions in the World Collection of Sugarcane and Related Grasses in Miami, FL, USA.**
(XLSX)

Table S3 **Sample name, species, group assignment, and subpopulation assignment of 1002 accessions genotyped in the World Collection of Sugarcane and Related Grasses in Miami, FL, USA.**
(XLSX)
